# Remembering a warm day: daytime temperature influences nighttime hypocotyl growth in Arabidopsis

**DOI:** 10.1093/plcell/koac085

**Published:** 2022-03-10

**Authors:** Sophie Hendrix

**Affiliations:** Assistant Features Editor, *The Plant Cell*, American Society of Plant Biologists, USA; Institute of Crop Science and Resource Conservation, University of Bonn, Bonn, Germany; Centre for Environmental Sciences, Hasselt University, Diepenbeek, Belgium

Extreme temperatures are generally detrimental to plant growth. However, mild temperature increases can actually favor the growth of certain plant organs through a process known as thermomorphogenesis. A classic example is the enhanced hypocotyl elongation in *Arabidopsis thaliana* seedlings exposed to elevated ambient temperatures. The transcription factor PHYTOCHROME INTERACTING FACTOR 4 (PIF4) functions as a central hub in this response and promotes hypocotyl growth by transcriptionally inducing auxin biosynthesis genes (Quint et al., 2016). One of the many players that regulate PIF4 expression or function is EARLY-FLOWERING 3 (ELF3). This protein is a component of the circadian clock evening complex, which serves as a transcriptional repressor of *PIF4*. Furthermore, ELF3 can physically interact with PIF4, preventing it from binding to its target genes (Casal and Balasubramanian, 2019). So far, research in this field has mainly focused on elucidating how these thermomorphogenic regulators influence hypocotyl growth in response to the current temperature. Now, **Germán Murcia and colleagues (****Murcia et al., 2022****)** have demonstrated that PIF4 and ELF3 also store information on temperatures to which the plant was previously exposed, enabling nighttime hypocotyl growth to respond to the preceding daytime temperature (see Figure).

**Figure:**
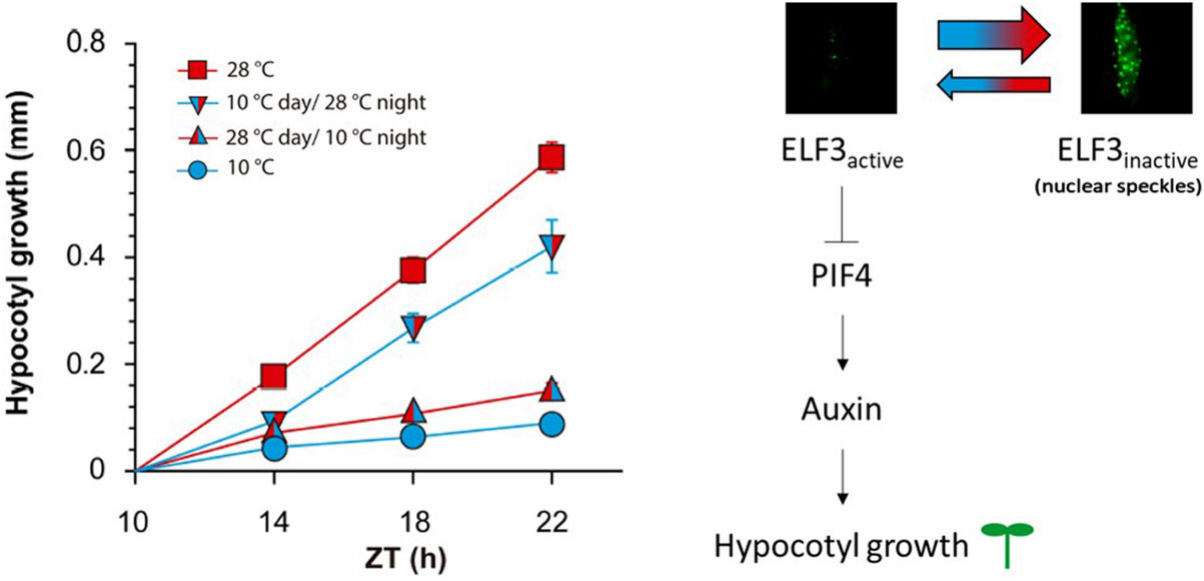
Nighttime hypocotyl growth in Arabidopsis is influenced by the current temperature as well as the preceding daytime temperature (left panel; adapted from Murcia et al. (2022), Figure 1; data represent the mean ± se of nine replicate boxes of seedlings; ZT: zeitgeber time). This short-term temperature memory relies on hysteresis in ELF3 and PIF4 dynamics, which respond rapidly to increasing temperatures but are less sensitive to subsequent cooling. PIF4 promotes hypocotyl growth by positively regulating auxin biosynthesis genes (right panel; figure credit: S. Hendrix; confocal images courtesy of Germán Murcia).

To demonstrate the presence of this short-term temperature memory, the authors grew Arabidopsis seedlings under short-day conditions and exposed them to all possible combinations of 2 day and night temperatures (10°C and 28°C). Measurements of hypocotyl length at different time points during the night revealed that hypocotyls of seedlings transferred from 10°C to 28°C at the beginning of the night grew less during the night than those of seedlings continuously kept at 28°C. Conversely, seedlings transferred from 28°C to 10°C showed enhanced nighttime hypocotyl growth compared with seedlings that were maintained at 10°C during both day and night (see Figure, left panel).

To further unravel how daytime temperature affects nighttime growth, responses of hypocotyl elongation to different combinations of day and night temperatures were monitored in different mutant backgrounds. Short-term temperature memory was disturbed in mutants of several known thermomorphogenic regulators including PIF4 and another transcription factor, ELONGATED HYPOCOTYL 5 (HY5). Confocal imaging of plants expressing PIF4 and HY5 reporter constructs indicated that nuclear levels of both proteins accounted for 80% of the observed growth rate variability. Using luminescence assays, the authors demonstrated that temperature-induced effects on nuclear PIF4 protein levels were largely driven by changes in *PIF4* promoter activity. The transfer of seedlings from 10°C to 28°C at the beginning of the night caused a rapid increase in nuclear PIF4 levels. However, the positive effect of a warm daytime temperature on PIF4 levels was not reversed by a cold night. Such an asymmetric response is typical for hysteresis, a phenomenon during which the relationship between the change in a variable (temperature) and its consequence (PIF4 levels) not only depends on the size of the change but also its direction.

As ELF3 is a known regulator of *PIF4* expression, the authors investigated the involvement of this clock protein in the observed temperature memory. ELF3 undergoes a phase transition in response to warm temperatures, forming speckles in the nucleus via its predicted prion-like domain (Jung et al., 2020). Murcia et al. (2022) demonstrated that ELF3 speckles formed in response to warm temperatures in the afternoon but not in the morning. Promoter activity analysis of ELF3-regulated genes identified a negative correlation between speckle formation and ELF3 activity. Interestingly, the ability of PIF4 to store information on daytime temperature was abolished in the *elf3* mutant background, indicating that ELF3 functions upstream of PIF4 in establishing short-term temperature memory. This was further supported by the observation that ELF3 speckle formation also showed a hysteretic pattern, with a high sensitivity to increasing temperatures and a much lower responsivity to cooling.

Taken together, Murcia et al. (2022) elegantly demonstrated that ELF3 and PIF4 dynamics show a hysteretic pattern that enables hypocotyl cells to remember previous exposure to warm temperatures. As thermomorphogenesis also takes place in many crop species, an improved understanding of its underlying mechanisms might facilitate the development of strategies to improve crop production in light of global warming.
